# Wnt Activation by Wild Type and Mutant Myocilin in Cultured Human Trabecular Meshwork Cells

**DOI:** 10.1371/journal.pone.0044902

**Published:** 2012-09-13

**Authors:** Xiang Shen, Hongyu Ying, Beatrice Y. J. T. Yue

**Affiliations:** Department of Ophthalmology and Visual Sciences, University of Illinois at Chicago College of Medicine, Chicago, Illinois, United States of America; Universidade Federal do Rio de Janeiro, Brazil

## Abstract

**Background:**

Myocilin is a gene linked to the most prevalent form of glaucoma, a major blinding disease. The trabecular meshwork (TM), a specialized eye tissue, is believed to be involved, at least in part, in the development of glaucoma. The Pro^370^ to Leu (P370L) mutation of myocilin is associated with severe glaucoma phenotypes and Gln^368^ stop (Q368X) is the most common myocilin mutation reported. Myocilin, upon overexpression, has been shown to induce phenotypes that include a loss of actin stress fibers, an increase in the cAMP level and protein kinase A (PKA) activity, as well as a reduction in the RhoA activity. We examined herein whether Wnt signaling pathway is involved in the myocilin phenotypes and whether P370L and Q368X mutants also display biological effects similar to those of the wild type myocilin.

**Methodology/Principal Findings:**

Wild type myocilin, when transfected into cultured human TM cells, induced a loss of actin stress fibers as judged by phalloidin staining. Such a loss was averted by treatment of secreted Frizzled-related protein 1 (sFRP1), an inhibitor of Wnt signaling. Consistent with the notion that Wnt pathway mediates the myocilin phenotype, Wnt activation was demonstrated by TOP/FOP-Flash reporter assays. Treatment of human TM cells of a Wnt activator, SB216763, as well as transfection of myocilin P370L and Q368X mutants all resulted in actin stress fiber loss, PKA activation and RhoA inactivation. The PKA elevation was obviated by the sFRP1 treatment, indicating that Wnt signaling was upstream that of PKA.

**Conclusions/Significance:**

The present study demonstrated that following forced expression of wild type myocilin, Wnt was activated, triggering in turn other myocilin-related alterations. P370L and Q368X mutations induced similar phenotypes, suggesting one possible mechanism how the mutants may lead to TM cell damage and pathology.

## Introduction

Glaucoma, one of the leading causes of irreversible blindness worldwide, is characterized by progressive loss of retinal ganglion cells and the accompanying axons, as well as cupping of the optic nerve head [1]. Primary open-angle glaucoma (POAG), the most common form of glaucoma, is frequently associated with elevated intraocular pressure (IOP) [2]. The IOP is controlled by a balance between the production and outflow of the aqueous humor contained in the anterior chamber of the eye. The trabecular meshwork (TM), a specialized tissue located next to the cornea, is the major site for regulation of the aqueous humor outflow [3], [4]. It is composed of layers of trabecular beams made up of extracellular matrix (ECM) elements. TM cells that cover the beams display an endothelial cell-like morphology and lining property but are of a unique cell type [4]. They are avid phagocytes [5], possess contractile and migratory apparatus [6], and have the capacity to produce ECM elements [4], [7]. It is believed that changes in the TM cell activities, cytoskeletal structure, cell-matrix and cell-cell adhesion, and/or the quantity and composition of the ECM may all produce adverse effects on the outflow pathway, leading to IOP elevation and ultimately glaucoma [7]–[9].

Recent studies have revealed that POAG is genetically heterogeneous, caused by a number of susceptibility genes and environmental factors [10], [11]. Myocilin (GLC1A) is the first gene identified for both juvenile- and adult-onset POAG [12]. More than 70 myocilin mutations have been found in POAG families. Glaucoma patients with myocilin mutation tend to have high IOP. Among the various myocilin mutations, Pro370Leu (P370L) mutation is responsible for one of the most severe glaucoma phenotypes and Gln368Stop (Q368X) is the most common mutation reported in POAG patients [13], [14].

Myocilin was initially identified as a 57−/55-kilodalton (kDa) protein secreted into the media of TM cultures after induction with glucocorticoids such as dexamethasone [15]. The myocilin mRNA and protein are present in a variety of ocular and nonocular tissues including the retina and the TM [14]. That myocilin expression can be induced dramatically by dexamethasone has been shown to be a distinct feature of TM cells [15], [16]. Myocilin, localized to both intracellular and extracellular sites in the TM cells and tissue [17], is speculated to have diverse functions.

When upregulated, the wild type myocilin may lead to pathology, as is observed in cases of corticosteroid glaucoma [18]. Earlier studies from our laboratory showed that overexpressing wild type myocilin intracellularly [19] by transfection or by protein transduction [20] in cultured human TM cells resulted in a loss of actin stress fibers and focal adhesions. Cell adhesion to fibronectin and cell spreading were also compromised [19]. These myocilin-induced events were further shown to be mediated via Rho GTPase and adenosine 3′,5′-cyclic monophosphate (cAMP)/protein kinase A (PKA) signaling. The cAMP level and PKA activity were elevated, and the downstream the RhoA activity was reduced [21].

Recombinant myocilin protein has recently been shown to interact with secreted inhibitors of Wnt signaling, secreted Frizzled-related protein 1 (sFRP1), sFRP3 and several other Frizzled receptors [22]. Wnts are a group of secreted, cysteine-rich glycoproteins [23]–[25]. The Wnt signaling can be divided into the β-catenin-dependent canonical and β-catenin-independent non-canonical pathways [23]–[25]. In the former, the Wnt pathway is activated when Wnts bind with a receptor complex consisting of Frizzled protein receptor and the low-density lipoprotein receptor-related proteins. Such activation induces breakdown of the destruction complex that consists of glycogen synthases kinase 3β (GSK-3β), whose major function is to phosphorylate β-catenin. In the absence of Wnt ligands, β-catenin is phosphorylated, and the phosphorylated β-catenin is constantly degraded in the cytoplasm. Upon Wnt activation, GSK-3β activity is inhibited, β-catenin is thus not phosphorylated, leading to β-catenin stabilization and nuclear translocation. In the nucleus, β-catenin binds to T-cell factor/lymphoid enhancing factor (Tcf/Lef) and regulates expression of target genes [23]–[26]. The non-canonical planar cell polarity pathway that activates small G proteins including Rac and Rho, and c-Jun N-terminal kinase [27], is involved in regulation of cytoskeletal organization and cell polarity. The second non-canonical pathway, Wnt/Ca^2+^, leads to release of intracellular Ca^2+^, and is involved in activation of protein kinase C and Ca^2+^/calmodulin-dependent protein kinase II. The Wnt/Ca^2+^ pathway has implications on cell proliferation and cell movement [28].

Wnt signaling pathways have been shown to play a role in eye diseases including retinal degeneration, cataract and congenital ocular malfunctions [29]. A possible involvement of Wnt signaling in the outflow system and glaucoma has also been reported [30]. While the underlying mechanism is still unclear, addition of exogenous sFRP1 to *ex vivo* perfusion cultured human eyes decreased outflow facility and increased the pressure, concomitant with reduced β-catenin levels. Expression of sFRP1 likewise induced IOP elevation in mice [30].

In light of the possible myocilin connection with the Wnt signaling pathway, we undertook the current study to determine whether Wnt signaling is a player in the myocilin-induced phenotypes. Results obtained indicated that Wnt was indeed activated upon myocilin overexpression. Interestingly, expression of P370L and Q368X mutant myocilins also induced phenotypes similar to those seen with the wild type.

## Results

### Aversion of the Actin and Focal Adhesion Phenotypes by Treatment of sFRP1

**Figure 1:**
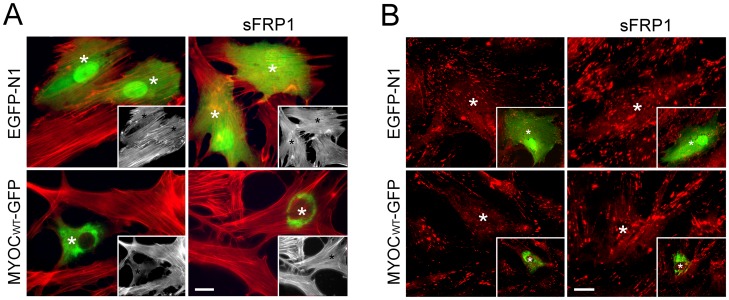
Treatment of sFRP1 averts the myocilin actin and focal adhesion phenotypes. TM cells were transfected with pEGFP-N1 (EGFP-N1, mock control) or pMyocilin_WT_-EGFP (MYOC_WT_-GFP) for 48 h, treated overnight with 50 nM sFRP1 and stained for actin (in red, **A**) or vinculin (in red, **B**). The transfected cells were marked by green fluorescence and/or white asterisks. Myocilin overexpression induced a loss of actin stress fibers (**A**) and vinculin-positive focal adhesions (**B**). The loss was averted by treatment of sFRP1. The staining was visualized using a Zeiss 100 M microscope. Insets in **A** show the actin stress fibers in same fields in black and white. The same transfected cells are indicated by black asterisks. Insets in **B** show the green transfected cells (white asterisks). Bar, 10 µm.

The myocilin construct pTarget-myocilin_WT_ or pMyocilin_WT_-EGFP was introduced into human TM cells. After transfection, the levels of myocilin protein, as judged by Western blotting, were approximately 10–20 fold higher than those in mock controls (data not shown). In agreement with findings from our previous studies [19], [21], the pMyocilin_WT_-EGFP-transfected green cells, compared to pEGFP-N1-transfected mock controls, showed an apparent loss of both actin stress fibers (Fig. 1A) and vinculin-positive focal adhesions (Fig. 1B).

To test whether Wnt signaling is involved in the myocilin phenotype, TM cells after myocilin transfection were treated with sFRP1, a Wnt signaling pathway inhibitor [25]. Results indicated that inhibition of Wnt pathway in pMyocilin_WT_-EGFP-transfected cells prevented, at least partially, the stress fiber dissolution and the focal adhesion reduction (Fig. 1), suggesting that Wnt signaling might mediate the myocilin-induced actin alteration.

### Effects of SB216763 on Human TM Cells

**Figure 2:**
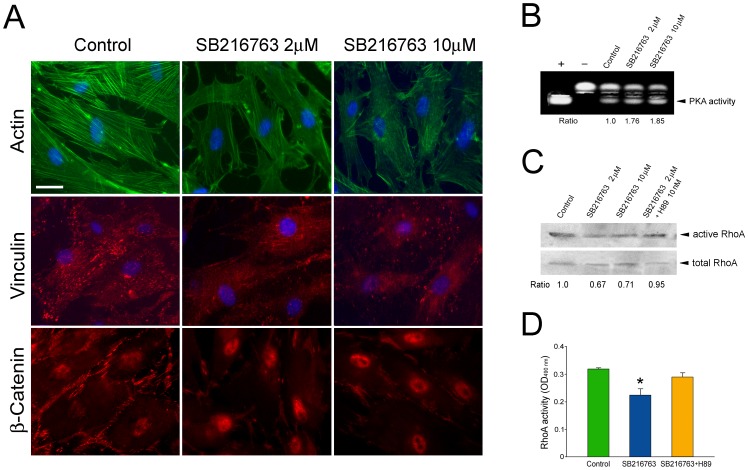
A. Actin (green), vinculin (red), and β-catenin (red) staining in normal human TM cells without (control) or with overnight treatment with 2 and 10 µM SB216763. Bar, 20 µm. **B**. PKA activity in TM cells without (control) or with treatment with SB216763. Equal amounts of protein lysates were subjected to PKA assays. Positive (+) and negative (-) controls were included. The non-phosphorylated (upper band) and the phosphorylated (lower band, arrowhead) substrates were resolved on agarose gels. The PKA activity, judged by the level of the phosphorylated substrate, in 2 (1.76±0.14, mean ± SD, n = 3) and 10 (1.85±0.15, n = 3) µM SB216763 treated cells (P<0.006 compared to control) was determined by densitometric analyses and normalized to that in untreated controls. **C**. RhoA activity in TM cells treated with 2 and 10 µM SB216763 or 2 µM SB216763 plus 10 nM H89, a PKA inhibitor. Cells untreated served as control. Active RhoA, measured by pull down assays, was normalized to total RhoA. The level of active RhoA was reduced by approximately 30% by treatment of SB216763. Its level however returned to normal when H89 was included in the treatment. **D**. RhoA activity as measured by G-LISA. The activity was lower in TM cells treated with 10 µM SB216763 (0.227±0.022 vs. 0.318±0.004 in control, mean ± SEM, n = 3). H89 treatment elevated the RhoA back to the control range (0.293±0.016, n = 3). *, P<0.001 compared to untreated control.

To support the notion that the myocilin phenotype was mediated via Wnt signaling, the effects of SB216763, a selective, cell permeable GSK-3 inhibitor, on normal human TM cells were investigated. This GSK-3 inhibitor has been commonly used to mimic the action of Wnt molecules to activate β-catenin [31]. As shown in Fig. 2, treatment with 2 and 10 µM of SB216763 triggered dissolution of actin stress fibers and loss of vinculin-positive focal adhesions (Fig. 2A). An enhanced nuclear staining of β-catenin was seen as expected in treated cells (Fig. 2A). SB216763 in addition caused an approximately 1.8-fold increase in the activity of PKA (Fig. 2B), a classical intracellular effector of the cAMP signaling. The level of active RhoA as measured by both pull down (Fig. 2C) and G-LISA (Fig. 2D) assays, on the other hand, was diminished. All these changes were reminiscent of those observed with myocilin overexpression. Furthermore, co-treatment of both SB216763 and H89, a PKA inhibitor, brought the RhoA activity back to normal (Figs. 2C and 2D), in keeping with our previous finding that cAMP/PKA activation was upstream of Rho inactivation [21].

### Wnt Activation upon Myocilin Transfection

For the analysis of β-catenin/Tcf/Lef signal activation, TOP-Flash/FOP-Flash luciferase assay has been the most commonly used [32]. The TOP-Flash construct contains two repeats of three optimal copies of the Tcf/Lef binding sites upstream of a thymidine kinase minimal promoter that directs transcription of a luciferase reporter gene. The FOP-flash, containing mutated Tcf/Lef binding sites, is used as a negative control.

Normal human TM cells were transfected with TOP-Flash or FOP-Flash plasmid. The cells in each well were co-transfected with pTarget-myocilin_WT_ or pTarget (control) to determine the myocilin effect, and also with pEGFP-N1 to control for variation in transfection efficiency. Results obtained revealed that in TM cells, the TOP-Flash response or the β-catenin/Tcf/Lef transactivation with or without myocilin transfection was marginal. The TOP-Flash/FOP-flash ratio was close to 1, contrasting to that seen in SW480 and Caco-2 human colorectal adenocarcinoma cell lines where the ratio was typically higher than 20 [33]. It is possible that similar to cell lines such as A549 and HeLa cells [33], TM cells are simply a cell type in which the constitutive Wnt signaling is less than modest. Furthermore, the thymidine kinase minimal promoter used in the TOP−/FOP-Flash constructs to drive luciferase expression is a relatively weak promoter. The low promoter activity plus the poor transfection efficiency (10–15%) in primary human TM cells might also explain for the low TOP-Flash readouts observed in the TM experiments.

**Figure 3:**
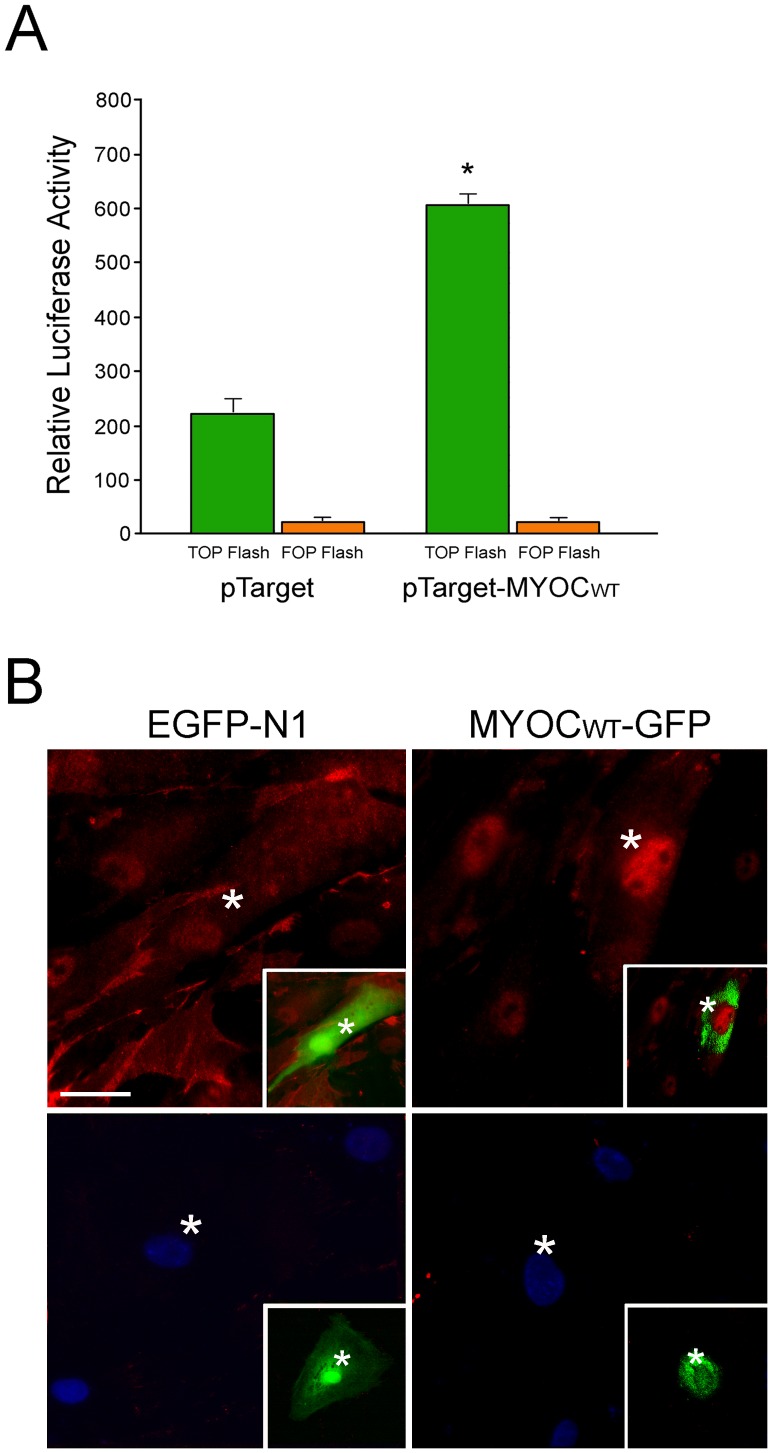
A. Myocilin expression activates Tcf/Lef-dependent transcription in Caco-2 cells. Caco-2 cells were co-transfected with TOP- or FOP-Flash reporter construct, and pTarget-myocilin_WT_ (pTarget-MYOC_WT_) or pTarget plasmid for 48 h. A pEGFP-N1 vector was also co-transfected for standardization. Luciferase activity measured was normalized to the GFP reading. Three experiments were performed in triplicate. Results (mean ± SEM) from a representative experiment are shown. *, P<0.001 compared to pTarget control. **B**. β-catenin (red) staining (top panel) in human TM cells after transfection with pEGFP-N1 (EGFP-N1, mock control) or pMyocilin_WT_-EGFP (MYOC_WT_-GFP). Minimal staining was observed when normal mouse IgG was used in place of primary anti-β-catenin (bottom panel) in the procedure. Insets highlight the green transfected cells (white asterisks) in the same fields. Nuclei were stained by DAPI in blue. Bar, 20 µm.

Caco-2 cells, as mentioned above, have been used in the literature for TOP-Flash reporter assays [34], [35]. Experiments were thus repeated using Caco-2 cells as surrogates to determine the Wnt activation upon myocilin transfection. An approximately 2.7 fold, significant (P<0.001 compared to pTarget control, n = 3) increase in the Lef/Tcf/Wnt activation following co-transfection of myocilin construct was found in Caco-2 cells (Fig. 3A). The TOP-Flash to FOP-Flash ratios were 55.3±3.4 and 20.0±2.9, respectively, in pTarget-myocilin_WT_- and pTarget-co-transfected samples. In agreement with the Wnt activation, the nuclear β-catenin staining was enhanced in myocilin-overexpressing TM cells compared to GFP mock transfected and non-transfected controls (Fig. 3B).

### Mutants Display Phenotypes Similar to the Wild Type Myocilin

**Figure 4:**
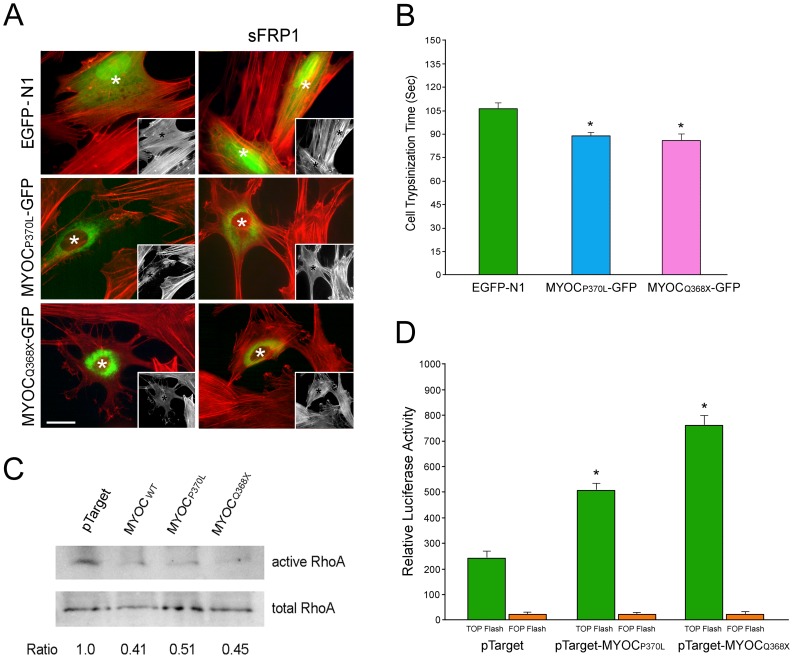
A. Actin (red) staining in pEGFP-N1 (EGFP-N1)-, pMyocilin_P370L_-EGFP (MYOC_P370L_-GFP)-, and pMyocilin_Q368X_-EGFP (MYOC_Q368X_-GFP)-transfected TM cells without or with subsequent overnight treatment of sFRP1. Insets show the same field in black and white. Transfected cells are marked by green fluorescence, or white or black (in insets) asterisks. Bar, 20 µm. **B**. The trypsinization time (mean ± SEM) needed for pEGFP-N1 (EGFP-N1)-, pMyocilin_P370L_-EGFP (MYOC_P370L_-GFP)-, and pMyocilin_Q368X_-EGFP (MYOC_Q368X_-GFP)-transfected TM cells to become refractile. Asterisk indicates that the trypsinization time for myocilin mutant transfectants was significantly (P<0.0001, n = 30) lower than that of GFP controls. **C**. GTP-bound active RhoA in pTarget-, pTarget-myocilin_WT_ (MYOC_WT_)-, pTarget-myocilin_P370L_ (MYOC_P370L_)- and pTarget-myocilin_Q368X_ (MYOC_Q368X_)-transfected TM cells. Pull down assays were performed in duplicates to determine the RhoA activity. The amount of the active or GTP-bound RhoA was normalized against the total amount in cell lysates and expressed as mean ± SD relative to that in pTarget control. The RhoA activity upon myocilin wild type (0.41±0.04, n = 2) and mutant transfection (0.51±0.03 for P370L; 0.45±0.04 for Q368X, n = 2) was found to be significantly reduced (P<0.0001) compared to controls. Experiments were repeated two times with similar results. **D.** Top−/Fop-Flash assays. Caco-2 cells were co-transfected with TOP- or FOP-Flash reporter construct, pTarget-myocilin_P370L_, pTarget-myocilin_Q368X_, or pTarget, as well as pEGFP-N1. Luciferase activity (mean ± SEM, n = 8) was measured post transfection and was normalized to the GFP level. *, P<0.001 compared to the pTarget control.

Transfection of normal human TM cells with pMyocilin_P370L_-EGFP and pMyocilin_Q368X_-EGFP, similar to that observed with the wild type, resulted in a loss of actin stress fibers (Fig. 4A). To examine the cell-matrix cohesiveness, TM cells were subjected to trypsin sensitivity tests as previously described [21]. It was found that the cells, upon myocilin mutant transfection, became more sensitive to trypsinization. The trypsinization time (Fig. 4B) needed for cells to round up was significantly (P<0.0001) shorter for P370L (88.9±1.5 s, n = 30) and Q368X (85.3±3.7 s, n = 30) myocilin mutant transfectants than that for mock controls (106.0±3.1 s, n = 30).

Pull down assays were carried out to measure the amounts of active RhoA in mock-, pTarget-myocilin_P370L_ -, and pTarget-myocilin_Q368X_-transfected cells. The level of GTP-bound or active RhoA in myocilin mutant transfectants was lower (Fig. 4C) than that in controls. Quantitation by densitometry and normalization to the total RhoA revealed that the RhoA activity upon P370L and Q368X transfection was significantly (P<0.0001) reduced compared to the mock control.

Both P370L and Q368X mutants activated Wnt signaling in Caco-2 cells (Fig. 4D), as indicated by the higher TOP-Flash to FOP-Flash ratios in pTarget-myocilin_P370L_ (45.8±7.5, n = 8)- and pTarget-myocilin_Q368X_ (67.3±9.2, n = 8)-co-transfected samples compared to the pTarget (19.9±3.3, n = 8) controls. The ratios were significantly (P<0.0001) different.

### PKA Activity

**Figure 5:**
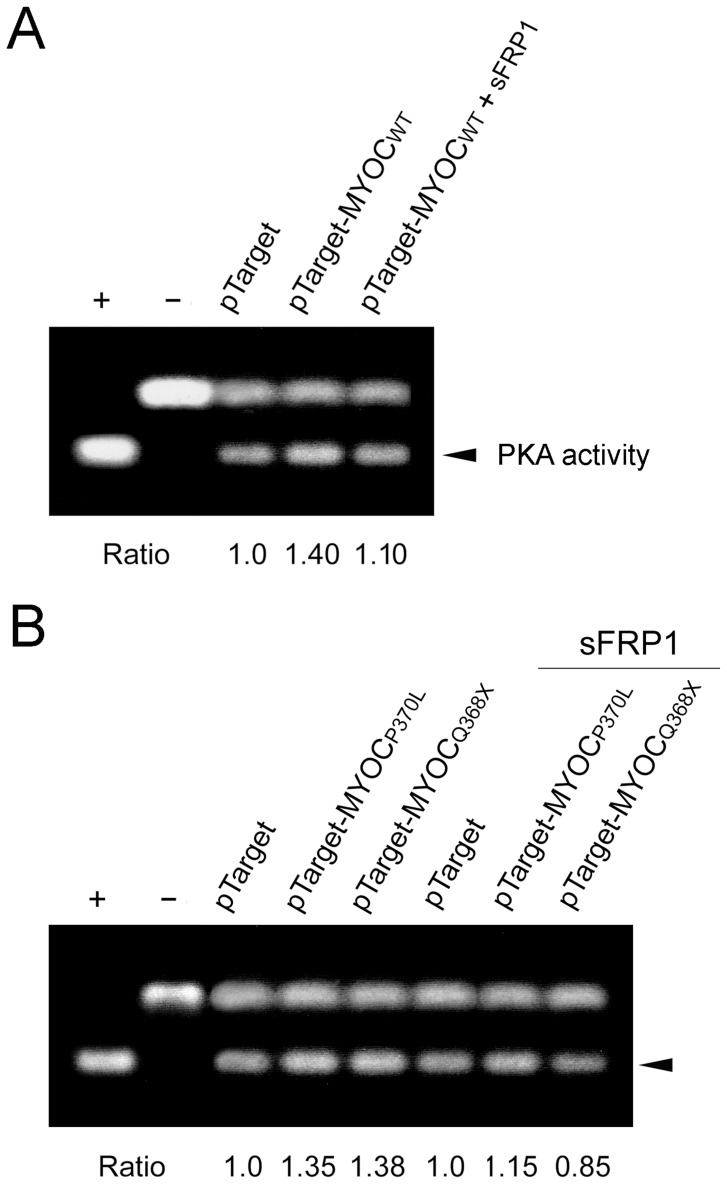
Effects of sFRP1 on PKA activity in TM cells. **A**. Cells were transfected with pTarget and pTarget-myocilin_WT_ (pTarget-MYOC_WT_) for 48 h. One set of samples was treated overnight with sFRP1 and the other was untreated. Lysates collected were subjected to PKA assay as described in Fig. 2B. **B**. Cells transfected with pTarget, pTarget-myocilin_P370L_ (pTarget-MYOC_P370L_) and pTarget-myocilin_Q368X_ (pTarget-MYOC_Q368X_) were untreated or treated overnight with sFRP1. Lysates were subjected to PKA assays. The PKA activity (arrowhead) was determined by densitometric analyses. Results were expressed as ratios relative to the pTarget control. Experiments were repeated 3 times. Data from one representative experiment are presented.

To determine whether Wnt signaling is up- or down-stream that of cAMP/PKA, human TM cells transfected with pTarget-myocilin_WT_ were treated with sFRP1. PKA assays showed that the enzyme activity (Fig. 5A) was elevated by approximately 40% (P<0.016), as seen previously [21] by forced expression of wild type myocilin (1.40±0.13, mean ± SD, n = 3) but the activity was reduced back to normal when Wnt activation was inhibited (1.10±0.12, n = 3, P>0.05 compared to pTarget control). Furthermore, PKA assays revealed that the kinase activity (Fig. 5B) was significantly (P<0.05) increased in myocilin P370L (1.35±0.15, n = 3) and Q368X (1.38±0.21, n = 3) mutant-expressing TM cells. Similar to that observed with the wild type, the PKA elevation in the mutant transfectants (1.15±0.12 and 0.85±0.10 respectively, P>0.05) was obviated by treatment of sFRP1, signifying again that Wnt pathway was upstream of PKA signaling.

## Discussion

The present study demonstrated that wild type myocilin, when overexpressed at a moderate level, activated the Wnt signaling pathway, which in turn mediated downstream events such as loss of actin stress fibers and elevation of the PKA activity. The notion of Wnt mediation is supported by observations that a Wnt inhibitor, sFRP1, was able to avert the myocilin-induced alterations (Figs. 1 & 5). Moreover, treatment of SB216763, a Wnt-enhancing agent, led to myocilin phenotypes including loss of actin stress fibers and focal adhesions, increase of PKA activity, as well as inhibition of RhoA activity (Fig. 2). The Wnt activation by myocilin expression, as evidenced by TOP−/FOP-Flash luciferase assays (Fig. 3) in the current study, is in agreement with an earlier observation that the β-catenin immunostaining was enhanced, compared to control littermates, in the angle tissues (including TM) of transgenic mice produced using bacterial artificial chromosome DNA containing full length human myocilin gene [22].

The Wnt signaling is a major intracellular pathway that plays important roles in diverse biological processes including cell proliferation, differentiation, development, angiogenesis and inflammation [23], [24], [36], [37]. Extensive studies of the Wnt pathway have been documented in the areas of cancer, stem cells and neural development [23], [24], [26], [35]. The Wnt genes can signal through a β-catenin/Tcf/Lef-dependent canonical pathway as well as two β-catenin-independent, non-canonical pathways. In the case of myocilin, it seems that the canonical pathway was a player since sFRP1 has been indicated in the literature to be an inhibitor to the Wnt canonical pathway [38]. Moreover, transactivation of Tcf/Lef transcription factors in the canonical pathway by myocilin was demonstrated by TOP/FOP-Flash assays (Fig. 3). sFRP1 and other sFRPs however have also been shown to exert their effects via both canonical and non-canonical signaling [25], [38]. There have been many examples more recently noting that individual modulators of Wnt signaling can be involved in several interwoven branches [36]. The additional involvement of non-canonical pathways in the myocilin-induced event is hence a distinct possibility.

The Wnt activation depends on Wnt ligands and specific sets of receptors. The aspects of Wnt network activated may depend on the cell type, conditions and the cellular context such as local concentrations of Wnt individual elements [36]. Wnt/β-catenin signaling can additionally be activated via Wnt-independent mechanisms including G proteins [26]. The mechanism by which myocilin regulates the Wnt signaling is unclear. Myocilin has been shown to interact with sFRP1, sFRP3 and other Frizzled receptors [22]. Such direct interactions, along with other factors/events, may be involved in the Wnt activation upon myocilin overexpression.

The Wnt signaling is known to cross talk with other signaling mechanisms [39] including Notch, NF-κB, mTOR, transforming growth factor -β, protein kinase C [26] and PKA [40]–[43]. Among them, PKA has been shown in a number of investigations to act upstream of Wnt signaling, resulting in β-catenin stabilization either via phosphorylation on Ser 9 of GSK-3ß inhibiting thereby its activity [41], or by direct phosphorylation of ß-catenin on Ser 675 to inhibit ß-catenin degradation or promote its binding to CREB-binding protein [42]. PKA has also been noted in a few reports to act downstream of Wnt signaling. For instance, Wnt proteins such as Wnt1 and Wnt7a were found to activate adenyl cyclase, cAMP and PKA to induce myogenesis during embryogenesis [43]. Adding to this list, the present study also concluded that PKA was downstream of Wnt signaling since the heightened PKA activity in myocilin-expressing cells declined back to the normal level (Fig. 5) following treatment of Wnt inhibitor sFPR1.

**Figure 6:**
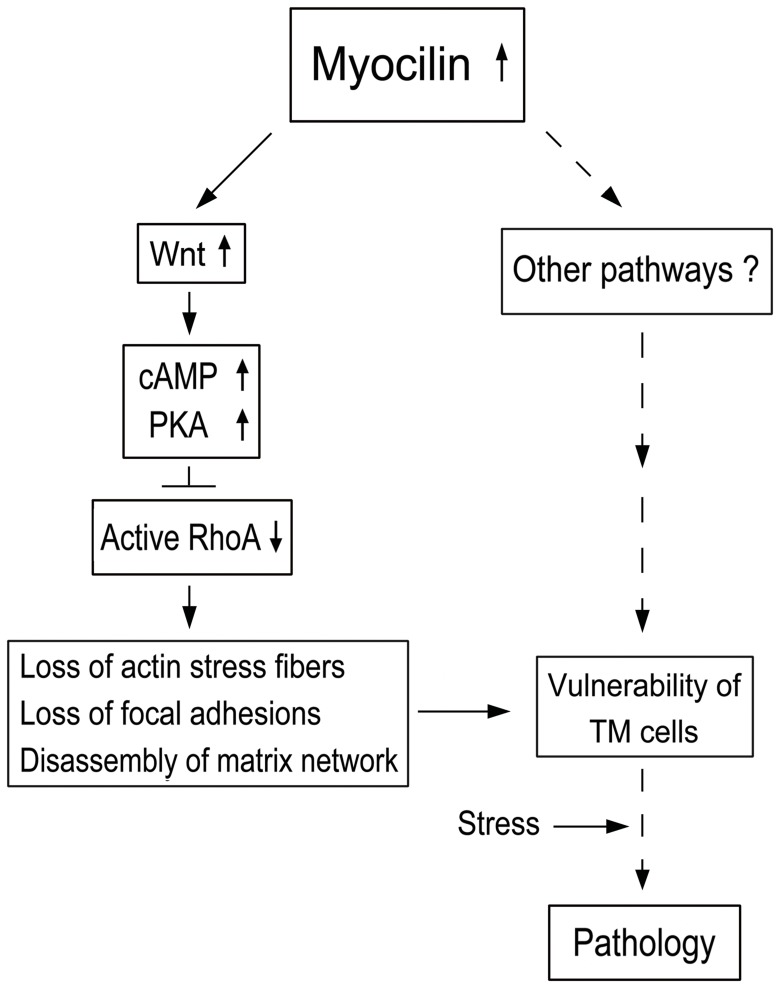
A schematic model of possible events in human TM cells triggered by upregulation of myocilin. Myocilin, when moderately upregulated, induces Wnt activation, and the downstream cAMP/PKA activation and RhoA inactivation, resulting in a loss of actin stress fibers and focal adhesions and disassembly of matrix network. These changes or other pathways may affect the integrity of TM cells, rendering them more susceptible to additional stress or challenge on a chronic, long-term basis, leading to pathologic consequences. Other intermediate mediators or pathways are yet to be identified. Dotted lines indicate steps that have not been investigated in the present work or our previous investigation [19]–[21].

Based on the current findings, a previously proposed schematic model can be modified as in Fig. 6. We surmise that moderately upregulated wild type myocilin activates Wnt signaling pathway, which leads to cAMP elevation, PKA activation and RhoA inactivation, and in turn triggers the loss of actin stress fibers and focal adhesions in TM cells, rendering them in a de-adhesive state. Through these steps and/or other pathways, the de-adhesion process on a long term chronic basis may leave the cells vulnerable to develop pathology upon additional stress. The involvement of the Wnt signaling demonstrated herein suggests an intervention possibility in rescuing the myocilin phenotypes.

In the *in vivo* TM system, the actin cytoskeleton system has been linked to regulation of the aqueous humor outflow [8], [9]. Experiments in living monkeys or perfusion organ cultures have shown that agents including cytochalasins, H-7, and latrunculins which perturb the actin structure increase the outflow facility. The increased flow is believed to result from the separation of cells from their neighboring cells and the ECM and the change in the overall TM geometry through cellular relaxation and contraction. A decrease in actin stress fibers and focal adhesions has also been found to occur upon treatment with Rho kinase (ROCK) inhibitor Y-27632 [44], and gene transfer of dominant negative RhoA [45] or C3 transferase [46] in TM cells. ROCK inhibitors have in addition been shown to decrease myosin light chain phosphorylation, which leads to relaxation of TM and Schlemm’s canal cells [47]. Currently, selective ROCK inhibitors such as AR-12286 are in clinical trials [48].

It is of note that experiments with monkey and perfusion organ culture were all short term, from a few hours to up to 7 days [8], [9]. A phase IIb, 28-day, safety and efficacy trial of topical AR-12286 has been completed [48] although its long term tolerability profile is still unknown. Glaucoma is a chronic, age-related disease. On a long term and persistent basis and especially because the TM cells *in situ* are continually subjected to stress from the aqueous flow and IOP fluctuations, it is conceivable that the altered actin cytoskeleton and impaired cell-matrix adhesiveness observed with overexpressed wild type myocilin would ultimately weaken the TM cells and destabilize the system. Cell loss and pathologic consequences may then ensue. The vulnerability of myocilin-transfected TM cells has been implicated by their susceptibility to apoptotic challenge [19]. Evidence was presented that anti-Fas treatment triggered a significantly higher level of apoptosis in myocilin transfectants than the mock controls [19].

Contrasting our results, Kwon et al [22] reported earlier that the myocilin action likely resembled non-canonical Wnt signaling since addition of recombinant human myocilin to cultures of human TM or mouse NIH 3T3 cells induced stress fiber formation and activated Rac1 without nuclear translocation of β-catenin [22]. It is of note that the experimental approaches used for myocilin studies, one by transfection for intracellular expression, and the other by addition of exogenous protein for extracellular presence [22], are utterly different. Wnt signaling seems to be activated in both scenarios but the downstream consequences are divergent. Myocilin is known to be localized both intra- and extra-cellularly. The key issue to be addressed next is how the intra- and extra-cellular myocilin signaling in TM tissue environment is intermingled and balanced in normal homeostasis and in stress conditions.

The activation of Wnt is also detected in cells expressing P370L and Q368X myocilin mutants (Fig. 4). These cells display all the wild type phenotypes: loss of actin stress fibers and focal adhesions, impairment in TM cell-matrix cohesiveness, activation of PKA, and reduction in the active RhoA level (Fig. 4). Since the 1–368 myocilin sequence is intact in both mutants, one may conclude that such a sequence is responsible for the myocilin phenotypes. On the other hand, the phenotypes may also be related to the retention of wild type, endogenous myocilin in the cells. P370L and Q368X mutants have been well documented to be secretion incompetent [49], [50]. They not only cannot be secreted themselves, but can also additionally block or suppress secretion of the endogenous myocilin. The net result would thus be intracellular accumulation of both mutant and wild type myocilins [49], [50], displaying the ensuing phenotypes.

Myocilin mutants including P370L and Q368X are detergent resistant [51]. Not being secreted, they accumulate intracellularly and have been shown to induce endoplasmic reticulum (ER) stress [52]–[54]. As a stress response, the cells activate the unfolded protein response, upregulating ER chaperones with the subsequent activation of caspases 12 and 3 and the expression of ER stress–initiated apoptotic transcriptional factor CHOP, leading to apoptosis [52]–[54]. Furthermore, P370L mutant has been reported to cause dysregulation of calcium channels and mitochondrial membrane depolarization [55]. Our demonstration of the myocilin phenotypes offers an additional mechanism as to how P370L and Q368X mutants may lead to cell vulnerability, increased cell susceptibility to stress, cell death, and ultimately pathology.

## Materials and Methods

### Cell Cultures

Normal human eyes were obtained from the Illinois Eye Bank (Chicago, IL). The TM tissues from donors 22, 27, 31, 39, 46, 52, and 55 years of age without any known ocular diseases were dissected and cultured as previously described [21] on Falcon Primaria flasks (BD Biosciences, Franklin Lakes, NJ) in complete media that contained Dulbecco’s modified Eagle’s minimum essential medium (DMEM), glutamine, 10% fetal bovine serum, 5% calf serum, and antibiotics. Second- or third-passaged cells were used for the study. In some experiments, normal human TM cells were treated overnight with 2 or 10 µM SB216763 (R&D Systems, Minneapolis, MN), a Wnt activator, in the absence of presence of 10 nM H89, a PKA inhibitor. To block the Wnt activation, cells after transfection were treated overnight with recombinant human sFRP-1 (50 nM, R&D Systems).

Caco2 cells obtained from American Type Cell Culture Collection (Manassas, VA) were cultured in complete media.

### Plasmid Construction

Plasmids pTarget-wild type myocilin (pTarget-myocilin_WT_) was constructed as previously described [21]. For pMyocilin_WT_-EGFP, polymerase chain reaction (PCR) was performed to amplify myocilin_WT_ and the *EcoRI/BamHI*-digested PCR product was cloned into pEGFP-N1 (BD Biosciences) at the corresponding sites. Sequencing was carried out to determine the proper orientation and confirm the construct sequences.

pMyocilin_P370L_-EGFP was generated by QuikChange II Site-Directed Mutagenesis Kit (Stratagene, La Jolla, CA). The sense primer sequence used for mutagenesis was: CCACGGACAGTTCCTGTATTCTTGGGGTGGC (C>T mutation is underlined).

The antisense primer sequence was the reverse. pMyocilin_Q368X_-EGFP was generated as follows: Myocilin_Q368X_ was amplified by PCR using forward primer: TCGAATTCCCACCATGGCTATGAGGTTCTTCTGT (*EcoRI* site is underlined) and reverse primer: TTGGATCCCGTCCGTGGTAGCCAGCTCCAGG.

(*BamHI* site is underlined) using pMyocilin_WT_-EGFP as template. PCR product was digested with *EcoRI* and *BamHI* and ligated in frame with same restriction enzymes digested pEGFP-N1 vector (Clontech) to generate plasmid pMyocilin_Q368X_-EGFP. Positive clone was sequenced to verify the construct. pTarget-myocilin_P370L_ and pTarget-myocilin_Q368X_ were similarly prepared.

pTarget-myocilin_WT_, pMyocilin_WT_-EGFP, and myocilin mutant constructs were introduced into human TM cells using FuGENE 6 (Roche, Indianapolis, IN) or Lipofectamine LTX (Life Technologies, Grand Island, NY) transfection reagent. TM cells were plated at 60% confluence 18 h before transfection. The vector:transfection reagent mixture was added to the cells for 48 h. As mock controls, TM cells were transfected in parallel with pTarget or pEGFP-N1 empty vector without the insert. The expression level of the transgene was examined by Western blotting [21] using a monoclonal [56] (a generous gift from Dr. M. Fautsch, Mayo Clinic) or a polyclonal (a generous gift from Dr. W.D. Stamer, Duke University) anti-myocilin antibody.

### Immunofluorescence and Actin Staining

Transfected human TM cells were plated onto Lab-Tek CC2 glass chamber slides (Nalge Nunc International, Naperville, IL). Cells were fixed in either ice cold methanol or paraformaldehyde-lysine-phosphate buffer, without or with permeabilization in 0.2% Triton X-100. After treatment of 3% H_2_O_2_ and blocking, the slides were incubated for 1 h with monoclonal anti-vinculin (Millipore, Billerica, MA) or anti-β-catenin (Santa Cruz Biotech., Santa Cruz, CA). Normal mouse IgG was used as a negative control. The cells were further incubated with Cy3-goat anti-mouse IgG. Slides were mounted in Vectashield (Vector Laboratories, Burlingame, CA) with DAPI (4′,6′-diamidino-2-phenylindole dihydrochloride). The staining was examined under a Leica confocal or a Zeiss 100 M microscope.

The actin structure in nontransfected, mock transfected and myocilin construct-transfected cells was examined using rhodamine-phalloidin (Life Technologies). The actin structure in normal human TM cells after treatment of SB216763 was stained with Oregon Green 488-phalloidin (Life Technologies).

### Trypsin Sensitivity

Trypsin sensitivity was examined using a Zeiss live cell imaging system (Carl Zeiss MicroImaging, Thornwood, NY). Cells were transfected with pEGFP-N1, pMyocilin_WT_-EGFP, pMyocilin_P370L_-EGFP, or pMyocilin_Q368X_-EGFP for 48 h. The cells were washed with Versene and observed under the Zeiss system using a 20× objective. Images were captured every 15 s after the addition of 0.25% trypsin solution to the culture. Cell changes were monitored and the time (mean ± SD) needed for the cells to shrink, round up and break away from the culture plate was determined [21].

### Active RhoA

Active RhoA was measured with pull down assay [21] or with G-LISA assays for RhoA (G-LISA kit, Cytoskeleton Inc., Denver, CO) in the colorimetric detection format. Normal human TM cells were untreated or treated overnight with 2 or 10 µM SB216763. Another set of cells was also co-treated with 10 nM H89. The cells were lysed in lysis buffer (G-LISA kit, Cytoskeleton Inc.) containing protease inhibitors (Roche). The proteins in the lysate were quantified by Bradford protein assay using bovine serum albumin as a standard. Pull down assays (Rho activation kit, Cytoskeleton Inc.) were performed as previously described [21]. pTarget mock- and pTarget-myocilin_WT_-_,_ pTarget-myocilin_P370L_- or pTarget-myocilin_Q368XT_-transfected cells serum starved for 18 h were lysed. Lysates from transfected cells were mixed at 4°C with GST-Rhotekin bound to Sepharose beads for 1 h. The proteins bound to the beads were resolved by 12% SDS-PAGE and immunoblotted with anti-RhoA. Cell lysates preincubated with GTPγ S and GDP served as positive and negative controls respectively. Prior to incubation with the beads, aliquots were removed from samples for total RhoA. Amounts of active GTP-RhoA bound to GST-Rhotekin were normalized against amounts of total RhoA in the cell lysates and the myocilin transfectant data were compared to controls.

G-LISA was performed as per the manufacturer’s instructions. Briefly, the active GTP-bound form of Rho present in the cell lysate (25 µg total protein) was allowed to bind to the 96-well plate coated with Rhotekin RBD domain of Rho-family effector proteins. Following incubation with mouse monoclonal anti-RhoA antibody (Cytoskeleton Inc.) and horseradish peroxidase (HRP)-conjugated secondary antibody as well as detection by the HRP detection reagent, the amounts of active RhoA in samples were measured by the absorbance at 490 nm using a Tecan microplate reader. The Rho control protein and lysis buffer served as positive and negative controls respectively**.** Amounts of active GTP-RhoA in myocilin transfectants were compared to mock-transfected controls.

### PKA Assay

The PKA activity in equal protein aliquots of lysates from mock- and myocilin-transfected cells was assessed by the incorporation of phosphate in Leu-Arg-Arg-Ser-Leu-Gly peptide using the non-radioactive Peptag system (Promega, Madison, WI). Negative and positive controls were included and the PKA activity was determined by densitometric analyses [21].

### TOP/FOP-Flash Assays

Normal human TM and Caco-2 cells were co-transfected with TOP-Flash or FOP-Flash reporter plasmid (Millipore, Temecula, CA), pTarget-myocilin_WT_, pTarget-myocilin_P370L_, pTarget-myocilin_Q368X_, or pTarget, as well as pEGFP-N1 (1∶1∶0.5 ratio) for 48 h. The cells were harvested and lysed in Promega luciferase assay lysis buffer. Using the Promega luciferase assay kit, the luminescence from the TOP- or FOP-Flash was read on a luminometer (OPTOCOMP II, MGM instruments). GFP florescence was monitored using a Tecan microplate reader (excitation wavelength, 485 nm; emission wavelength, 535 nm). The TOP/FOP-Flash readings were normalized to the GFP reading. Experiments were performed in triplicates. At least 3 experiments were performed.

## References

[1] Quigley HA (2011) Glaucoma. Lancet 377: 1367–1377. 21453963 10.1016/S0140-6736(10)61423-7

[2] Kwon YH, Fingert JH, Kuehn MH, Alward WLM (2009) Primary open-angle glaucoma. N Engl J Med 360: 1113–1124. 19279343 10.1056/NEJMra0804630 PMC3700399

[3] Bill A (1975) The drainage of aqueous humor. Invest Ophthalmol Vis Sci 14: 1–3. 1110131

[4] Yue BYJT (1996) The extracellular matrix and its modulation in the trabecular meshwork. Surv Ophthalmol 40: 379–390. 8779084 10.1016/s0039-6257(96)80066-x

[5] Zhou L, Fukuchi T, Kawa JE, Higginbotham EJ, Yue BYJT (1995) Loss of cell-matrix cohesiveness after phagocytosis by trabecular meshwork cells. Invest Ophthalmol Vis Res 36: 787–795. 7706026

[6] Stumpff F, Wiederholt M (2000) Regulation of trabecular meshwork contractility. Ophthalmologica 214: 33–53. 10657743 10.1159/000027471

[7] Keller KE, Aga M, Bradley JM, Kelley MJ, Acott TS (2009) Extracellular matrix turnover and outflow resistance. Exp Eye Res 88: 676–682. 19087875 10.1016/j.exer.2008.11.023 PMC2700052

[8] Stamer WD, Acott TS (2012) Current understanding of conventional outflow dysfunction in glaucoma. Curr Opin Ophthalmol 23: 135–143. 22262082 10.1097/ICU.0b013e32834ff23e PMC3770936

[9] Tian B, Gabelt BT, Geiger B, Kaufman PL (2009) The role of the actomyosin system in regulating trabecular fluid outflow. Exp Eye Res 88: 713–717. 18793636 10.1016/j.exer.2008.08.008 PMC2694502

[10] Fingert JH (2011) Primary open angle genes. Eye 25: 587–595. 21562585 10.1038/eye.2011.97 PMC3171270

[11] Wang N, Chintala SK, Fini ME, Schuman JS (2001) Activation of a tissue-specific stress response in the aqueous outflow pathway of the eye defines the glaucoma disease phenotype. Nat Med 7: 304–309. 11231628 10.1038/85446 PMC1945815

[12] Stone EM, Fingert JH, Alward WL, Nguyen TD, Polansky JR, et al (1997) Identification of a gene that causes primary open angle glaucoma. Science 275: 668–670. 9005853 10.1126/science.275.5300.668

[13] Gong G, Kosoko-Lasaki O, Haynatzki GR, Wilson MR (2004) Genetic dissection of myocilin glaucoma. Hum Mol Genet 13: R91–102. 14764620 10.1093/hmg/ddh074

[14] Tamm ER (2002) Myocilin and glaucoma: facts and ideas. Prog Retin Eye Res 21: 395–428. 12150989 10.1016/s1350-9462(02)00010-1

[15] Nguyen T, Chen P, Huang W, Chen H, Johnson D, et al (1998) Gene structure and properties of TIGR, an olfactomedin-related glycoprotein clones from glucocorticoid-induced trabecular meshwork cells. J Biol Chem 273: 6341–6350. 9497363 10.1074/jbc.273.11.6341

[16] Wentz-Hunter K, Shen X, Yue BYJT (2003) Distribution of myocilin, a glaucoma gene product, in human corneal fibroblasts. Mol Vis 9: 308–314. 12847420

[17] Ueda J, Wentz- Hunter K, Cheng EL, Fukuchi T, Abe H, et al (2000) Ultrastructural localization of myocilin in human trabecular meshwork cells and tissues. J Histochem Cytochem 48: 1321–1329. 10990486 10.1177/002215540004801003

[18] Mao W, Tovar-Vidales T, Yorio T, Wordinger RJ, Clark AF (2011) Perfusion-cultured bovine anterior segments as an ex vivo model for studying glucocorticoid-induced ocular hypertension and glaucoma. Invest Ophthalmol Vis Sci 52: 8068–8075. 21911581 10.1167/iovs.11-8133 PMC3208005

[19] Wentz-Hunter K, Shen X, Okazaki K, Tanihara H, Yue BYJT (2004) Overexpression of myocilin in cultured human trabecular meshwork cells. Exp Cell Res 297: 39–48. 15194423 10.1016/j.yexcr.2004.02.024

[20] Sakai H, Park BC, Shen X, Yue BYJT (2006) Transduction of TAT-fusion proteins into the human and bovine trabecular meshwork. Invest Ophthalmol Vis Sci 47: 4427–4234. 17003436 10.1167/iovs.06-0047

[21] Shen X, Koga T, Park BC, SundarRaj N, Yue BYJT (2008) Rho GTPase and cAMP/PKA signaling mediates myocilin induced alterations in cultured human trabecular meshwork cells. J Biol Chem 283: 603–612. 17984096 10.1074/jbc.M708250200 PMC2729092

[22] Kwon H-S, Lee H-S, Ji Y, Rubin JS, Tomarev SI (2009) Myocilin is a modulator of Wnt signaling. Mol Cell Biol 29: 2139–2154. 19188438 10.1128/MCB.01274-08 PMC2663295

[23] Jin T, Fantus IG, Sun J (2008) Wnt and beyond Wnt: multiple mechanisms control the transcriptional property of β-catenin. Cell Signal 20: 1697–1704. 18555664 10.1016/j.cellsig.2008.04.014

[24] Cadigan KM, Peifer M (2009) Wnt signaling from development to disease: insights from model systems. Cold Spring Harb Perspect Biol 1: a002881. 20066091 10.1101/cshperspect.a002881 PMC2742092

[25] Mii Y, Taira M (2011) Secreted Wnt “inhibitors” are not just inhibitors: regulation of extracellular Wnt by secreted Frizzled-related proteins. Develop Growth Differ 53: 911–923. 10.1111/j.1440-169X.2011.01299.x 21995331

[26] Luo J, Chen J, Deng Z, Luo X, Song W, et al (2007) Wnt signaling and human diseases: what are the therapeutic implications? Lab Invest 87: 97–103. 17211410 10.1038/labinvest.3700509

[27] Veeman MT, Acelrod JD, Moon RT (2003) A second canon. Functions and mechanisms of β-catenin-independent Wnt signaling. Dev Cell 5: 367–377. 12967557 10.1016/s1534-5807(03)00266-1

[28] Kühl M, Sheldahl LC, Park M, Miller JR, Moon RT (2000) The Wnt/Ca^2+^ pathway: a new vertebrate Wnt signaling pathway takes shape. Trends Genet 16: 279–283. 10858654 10.1016/s0168-9525(00)02028-x

[29] De Iongh RU, Abud HE, Hime GR (2006) Wnt/Frizzled signaling in eye development and disease. Front Biosci 11: 2442–2464. 16720326 10.2741/1982

[30] Wang WH, McNatt LG, Pang IH, Millar JC, Hellberg PE, et al (2008) Increased expression of the Wnt antagonist sFRP-1 in glaucoma elevates intraocular pressure. J Clin Invest 118: 1056–1064. 18274669 10.1172/JCI33871 PMC2242621

[31] Braeuning A, Buchmann A (2009) The glycogen synthase kinase inhibitor 3-(2,4-Dichlorophenyl)-4-(1-methyl-1H-indol-3-yl)-1H-pyrrole-2,5-dione (SB216763) is a partial agonist of the aryl hydrocarbon receptor. Drug Metabol Disposition 37: 1576–1580. 10.1124/dmd.109.027821 19448134

[32] Osada T, Chen M, Yang XY, Spasojevic I, Vandeusen JB, et al (2011) Antihelminth compound niclosamide downregulates Wnt signaling and elicits antitumor response in tumors with activating APC mutations. Cancer Res 71: 4172–4182. 21531761 10.1158/0008-5472.CAN-10-3978 PMC3117125

[33] Kuroda T, Rabkin SD, Martuza RL (2006) Effective treatment of tumors with β-catenin-T-cell factor activity by transcriptionally targeted oncolytic herpes simplex virus vector. Cancer Res 66: 10127–10134. 17047077 10.1158/0008-5472.CAN-06-2744

[34] Wang W, Liu H, Wang S, Hao X, Lin L (2011) A diterpenoid derivative 15-oxospiramilactone inhibits Wnt/β-catenin signaling and colon cancer cell tumorigenesis. Cell Res 21: 730–740. 21321609 10.1038/cr.2011.30 PMC3203668

[35] Logan CY, Nusse R (2004) The Wnt signaling pathway in development and disease. Annu Rev Cell Dev Biol 20: 781–810. 15473860 10.1146/annurev.cellbio.20.010403.113126

[36] Rao TP, Kuhl M (2010) An updated overview on Wnt signaling pathways. A prelude for more. Cir Res 106: 1798–1806. 10.1161/CIRCRESAHA.110.219840 20576942

[37] Dejana E (2010) The role of Wn signaling in physiological and pathological angiogenesis. Cir Res 107: 943–952. 10.1161/CIRCRESAHA.110.223750 20947863

[38] Yang Z, Liu G, Bollig-Fischer A, Haddad R, Tarca A, et al (2009) Methylation-associated silencing of SFRP1 with an 8p11–12 amplification inhibits canonical and non-canonical WNT pathways in breast cancers. Int J Cancer 125: 1613–1621. 19569235 10.1002/ijc.24518 PMC2735097

[39] Kikuchi A, Yamamoto, Sato A, Matsumoto S (2011) New insights into the mechanism of Wnt signaling pathway activation. Int Rev Cell Mol Biol 291: 21–71. 22017973 10.1016/B978-0-12-386035-4.00002-1

[40] Moon RT, Kohn AD, De Ferrari GV, Kaykas A (2004) Wnt and β-catenin signaling: diseases and therapies. Nature Rev Genetics 5: 689–699. 10.1038/nrg1427 15372092

[41] Fang X, Yu SX, Lu Y, Bast RC Jr, Woodgett JR, et al (2000) Phosphorylation and inactivation of glycogen synthase kinase 3 by protein kinase A. Proc Natl Acad Sci U S A. 97: 11960–11965. 10.1073/pnas.220413597 PMC17277 11035810

[42] Taurin S, Sandbo N, Qin Y, Browning D, Dulin NO (2006) Phosphorylation of β-catenin by cyclic AMP-dependent protein kinase. J Biol Chem 281: 9971–9976. 16476742 10.1074/jbc.M508778200

[43] Chen AE, Ginty DD, Fan CM (2005) Protein kinase A signaling via CREB controls myogenesis induced by Wnt proteins. Nature 433: 317–322. 15568017 10.1038/nature03126

[44] Honjo M, Tanihara H, Inatani M, Kido N, Yue BYJT, et al (2001) Effects of Rho-associated protein kinase inhibitor, Y-27632, on intraocular pressure and outflow facility. Invest Ophthalmol Vis Sci 42: 137–144. 11133858

[45] Vittitow JL, Garg R, Rowlette LLS, Epstein DL, O’Brien ET, et al (2002) Gene transfer of dominant-negative RhoA increases outflow facility in perfused human anterior segment cultures. Mol Vis 8: 32–44. 11889464

[46] Liu X, Hu Y, Filla MS, Gabelt BT, Peters DM, et al (2005) The effect of C3 transgene expression on actin and cellular adhesions in cultured human trabecular meshwork cells and on outflow facility in organ cultured monkey eyes. Mol Vis 11: 1112–1121. 16379023

[47] Zang M, Maddala R, Rao PV (2008) Novel molecular insights into RhoA GTPase-induced resistance to aqueous humor outflow through the trabecular meshwork. Am J Physiol 295: C1057–C1070. 10.1152/ajpcell.00481.2007 PMC2584993 18799648

[48] Chen J, Runyan SA, Robinson MR (2011) Novel ocular antihypertensive compounds in clinical trials. Clin Ophthalmol 5: 667–677. 21629573 10.2147/OPTH.S15971 PMC3104796

[49] Caballero M, Rowlette LL, Borras T (2000) Altered secretion of a TIGR/MYOC mutant lacking the olfactomedin domain. Biochim Biophys Acta 1502: 447–460. 11068187 10.1016/s0925-4439(00)00068-5

[50] Jacobson N, Andrews M, Shepard AR (2001) Non-secretion of mutant proteins of the glaucoma gene myocilin in cultured trabecular meshwork cells and in aqueous humor. Hum Mol Genet 10: 117–125. 11152659 10.1093/hmg/10.2.117

[51] Liu Y, Vollrath D (2004) Reversal of mutant myocilin non-secretion and cell killing: implications for glaucoma. Hum Mol Genet 13: 1193–1204. 15069026 10.1093/hmg/ddh128

[52] Joe MK, Sohn S, Hur W, Moon Y, Choi YR, et al (2003) Accumulation of mutant myocilins in ER leads to ER stress and potential cytotoxicity in human trabecular meshwork cells. Biochem Biophys Res Commun 312: 592–600. 14680806 10.1016/j.bbrc.2003.10.162

[53] Yam GH, Gaplovska-Kysela K, Zuber C, Roth J (2007) Aggregated myocilin induces Russell bodies and causes apoptosis: implications for the pathogenesis of myocilin-caused primary open-angle glaucoma. Am J Pathol 170: 100–109. 17200186 10.2353/ajpath.2007.060806 PMC1762699

[54] Zode GS, Kuehn MH, Nishimura DY, Searby CC, Mohan K, et al (2011) Reduction of ER stress via a chemical chaperone prevents disease phenotypes in a mouse model of primary open angle glaucoma. J Clin Invest 121: 3542–3553. 21821918 10.1172/JCI58183 PMC3163970

[55] He Y, Leung KW, Zhou YH, Ge J (2009) Pro370Leu mutant myocilin impairs mitochondrial functions in human trabecular meshwork cells. Mol Vis 15: 815–825. 19390644 PMC2672150

[56] Ezzat MK, Howell KG, Bahler CK, Beito TG, Loewen N, et al (2008) Characterization of monoclonal antibodies against the glaucoma-associated protein myocilin. Exp Eye Res 87: 376–384. 18674535 10.1016/j.exer.2008.07.002 PMC2866087

